# ASO Author Reflections: Persistent Pain After Breast Cancer Treatment, an Underreported Burden for Breast Cancer Survivors

**DOI:** 10.1245/s10434-024-15855-z

**Published:** 2024-07-21

**Authors:** Bo T. M. Strijbos, Loes Janssen, Adri C. Voogd, Willem A. R. Zwaans, Rudi M. H. Roumen, Adriana J. G. Maaskant-Braat

**Affiliations:** 1 Department of Surgical Oncology, Máxima Medical Center, Veldhoven, The Netherlands; 2 Department of Epidemiology, Maastricht University, Maastricht, The Netherlands; 3 NUTRIM School of Nutrition and Translational Research in Metabolism, Maastricht University, Maastricht, The Netherlands

## Past

In the past, many breast cancer survivors who had undergone surgery experienced persistent pain. Unfortunately, the exact pathophysiology of this pain, known as persistent pain after breast cancer treatment (PPBCT), was poorly understood and often not recognized. This lack of recognition hampered preoperative screening, counselling, and management of patients experiencing PPBCT. Additionally, past studies were frequently based on small sample sizes and lacked consistent definitions of PPBCT, leading to a wide range of reported prevalence rates.^1–4^ This inconsistency hindered the development and evaluation of standardized treatment protocols and left many survivors without adequate support or intervention strategies.

Furthermore, in the past, clinicians primarily concentrated on immediate postsurgical outcomes, neglecting the long-term management of chronic pain. Contradictory results from previous studies regarding potential risk factors further complicated efforts to understand the etiology and define risk groups, which is crucial to take preventive measures and develop effective treatment strategies for PPBCT.

## Present

Our current study represents a first step in addressing these gaps. By leveraging data from the Eindhoven Cancer Registry and including a large, unselected, population-based sample of 1048 women, we have been able to provide a more precise estimate of PPBCT prevalence and identify risk factors possibly associated with this condition. Our findings highlight the prevalence of PPBCT at 37.9% and underscore the importance of considering psychosocial factors, such as anxiety and depression, in pain management.^5^ The inclusion of multivariate analyses has allowed us to distinguish independent risk factors and better understand the complex interplay between patient, tumor and treatment characteristics. This comprehensive approach provides valuable insights that can inform clinical practice, improve patient outcomes, and address both the physiological and psychosocial aspects of persistent pain in breast cancer survivors.

## Future

Looking ahead, prospective studies are crucial to further refine our understanding of PPBCT and identify effective intervention targets. Future research should focus on the development and implementation of preventive strategies, including preoperative screening for psychosocial risk factors and the application of tailored interventions. Integrating patient-reported outcomes measures (PROMs) into routine preoperative assessments will be vital in capturing patient’s perspective and ensuring a patient-centered approach to pain management.

Although our study suggests that the type of treatment may not significantly influence the development of PPBCT, many questions remain. In particular, the association between radiotherapy and PPBCT needs further investigation to clarify whether radiotherapy contributes to the development of persistent pain. Therefore, prospective research is necessary to validate our findings and to further explore the complex factors contributing to PPBCT.

Additionally, exploring the efficacy of regional anesthesia, multimodal pain management strategies, and preventive behavioral interventions will be key in mitigating the burden of PPBCT. The goal is to establish evidence-based protocols that not only prevent the onset of chronic pain, but also provide effective relief for those already affected, ultimately enhancing the quality of life for breast cancer survivors.
